# Flavonoid-Labeled Biopolymer in the Structure of Lipid Membranes to Improve the Applicability of Antioxidant Nanovesicles

**DOI:** 10.3390/pharmaceutics16010141

**Published:** 2024-01-20

**Authors:** Patrick D. Mathews, Gabriella S. Gama, Hector M. Megiati, Rafael R. M. Madrid, Bianca B. M. Garcia, Sang W. Han, Rosangela Itri, Omar Mertins

**Affiliations:** 1 Laboratory of Nano Bio Materials (LNBM), Department of Biophysics, Paulista Medical School, Federal University of Sao Paulo, Sao Paulo 04023-062, Brazil; patrick.mathews@unesp.br (P.D.M.); gabgamasantos@gmail.com (G.S.G.); hectormegiati@gmail.com (H.M.M.); rafael.madrid@unifesp.br (R.R.M.M.); 2 Institute of Biosciences, Sao Paulo State University, Botucatu 18618-689, Brazil; 3 Interdisciplinary Center for Gene Therapy, Paulista Medical School, Federal University of Sao Paulo, Sao Paulo 04023-062, Brazil; bianca.bonetto@unifesp.br (B.B.M.G.); sang.han@unifesp.br (S.W.H.); 4 Applied Physics Department, Institute of Physics, University of Sao Paulo, Sao Paulo 05508-900, Brazil; itri@if.usp.br

**Keywords:** antioxidant, cubic-hexagonal topology, flavonoids, liposome, giant vesicle, liquid crystalline phase, SAXS

## Abstract

Nanovesicles produced with lipids and polymers are promising devices for drug and bioactive delivery and are of great interest in pharmaceutical applications. These nanovesicles can be engineered for improvement in bioavailability, patient compliance or to provide modified release or enhanced delivery. However, their applicability strongly depends on the safety and low immunogenicity of the components. Despite this, the use of unsaturated lipids in nanovesicles, which degrade following oxidation processes during storage and especially during the proper routes of administration in the human body, may yield toxic degradation products. In this study, we used a biopolymer (chitosan) labeled with flavonoid (catechin) as a component over a lipid bilayer for micro- and nanovesicles and characterized the structure of these vesicles in oxidation media. The purpose of this was to evaluate the in situ effect of the antioxidant in three different vesicular systems of medium, low and high membrane curvature. Liposomes and giant vesicles were produced with the phospholipids DOPC and POPC, and crystalline cubic phase with monoolein/DOPC. Concentrations of chitosan–catechin (CHCa) were included in all the vesicles and they were challenged in oxidant media. The cytotoxicity analysis using the MTT assay (3-(4,5-dimethyl-2-thiazolyl)-2,5-diphenyl-2H-tetrazolium bromide) revealed that concentrations of CHCa below 6.67 µM are non-toxic to HeLa cells. The size and zeta potential of the liposomes evidenced the degradation of their structures, which was minimized by CHCa. Similarly, the membrane of the giant vesicle, which rapidly deteriorated in oxidative solution, was protected in the presence of CHCa. The production of a lipid/CHCa composite cubic phase revealed a specific cubic topology in small-angle X-ray scattering, which was preserved in strong oxidative media. This study demonstrates the specific physicochemical characteristics introduced in the vesicular systems related to the antioxidant CHCa biopolymer, representing a platform for the improvement of composite nanovesicle applicability.

## 1. Introduction

The development of biofunctional materials based on biopolymers and lipids has been of great scientific and technological interest in recent years [1,2,3,4]. Chitosan consists of a polysaccharide of natural origin, and is biodegradable, non-toxic, non-antigenic, biocompatible and has antimicrobial activities [5,6,7]. These properties make chitosan a polymer of real applicability in association with liposomes, especially considering the improvement of characteristics such as stability, especially in the gastrointestinal tract, and the retention of encapsulated material and specificity for biomedical applications when bioadhesion is required [8,9,10,11].

The association of chitosan with liposomes has been proposed in the development of drug and vaccine carrier materials [12,13] as well as for nutraceuticals and nutritional compound preservation and prolonged release [14,15]. Previously, we have studied the structure of uni- and multilamellar liposomes with chitosan coating the membrane of the phospholipids internally and externally [16,17]. In addition, giant vesicles containing chitosan have been produced by a modification of the known electroformation method, thus allowing the incorporation of the polysaccharide over the lipidic membrane [18]. More recently, studies reporting the inclusion of chitosan and a chitosan derivative in liquid crystalline bicontinuous cubic phases and cubosomes have evidenced the potential of the polysaccharide in providing specific characteristics to the nanoparticles for improvement in bioactive delivery applications [19,20].

Moreover, the modification of chitosan has been considered in the improvement of advantageous properties for more specific applications [21]. A focused approach to enhance the functionality of chitosan is the chemical binding of active molecules from natural plant extracts [22]. Among them are flavonoids, which are known as efficient antioxidants that neutralize free radicals, preventing damage caused by oxidative stress [23]. In fact, flavonoids have been used as anti-inflammatory, anti-microbial and anti-cancer agents [24,25,26].

Free radicals are closely associated with reactive oxygen species (ROS) that cause aging and cell death [27]. ROS are produced by cellular metabolism, especially in degenerative processes associated with diseases, and their performance can be reduced, involving various antioxidant systems. Antioxidant substances reduce hydrogen radical (H^+^), superoxide anion (·O2^−^), hydroxyl radical (·OH^−^) and hydrogen peroxide (H_2_O_2_), hence protecting cells from the damage generated by these radicals. Many complexes containing thiamine, riboflavin, ascorbic acid, sinapic acid, *p*-coumaric acid and caffeic acid are used as antioxidants. However, these complexes are quickly metabolized and eliminated from the human body. Therefore, the development of a compound or system with high antioxidant activity remains a challenge to be overcome.

Various modifications of chitosan have been described. To produce chitosan with antioxidant properties, flavonoids and carotenoids were linked to the polysaccharide and the antioxidant properties were fully characterized [22,28]. However, chitosan presents aggregation processes at pH above 6, which alters the physicochemical properties, often making its application difficult.

Although the relative efficacy of chitosan-coated liposomes for drug delivery has been demonstrated, few studies have considered the potential of this same system as an antioxidant agent, using modified chitosan. Since the chemical binding of antioxidative compounds in chitosan was performed with relative success, an effective antioxidant lipid–chitosan system remains to be developed. Previously, we have labeled chitosan with gallic acid and performed studies using giant unilamellar vesicles (GUVs) produced with 1-palmitoyl-2-oleoyl-glycero-3-phosphocholine (POPC) lipid. We evidenced that the phenolic acid reduced the hydroperoxidation of the lipids when dissolved in the GUV solution and also when placed directly over the lipid membrane [29]. In the present study, we investigated the effect of chitosan partially modified by chemically linked catechin, a natural flavonoid with profitable antioxidant properties and with higher scavenging of ROS, if compared to gallic acid. The biomacromolecule was included in the structure of three different lipidic vesicular systems: liposomes, giant vesicles and liquid crystalline bicontinuous cubic phase, of a well-known medium, low and very high membrane curvature, respectively, and all were subjected to oxidative solutions. This study evaluated the stability of composite lipid–chitosan–catechin vesicular systems in oxidative media aiming to contribute to the development of antioxidant strategies for drug and nutraceutical compounds.

## 2. Materials and Methods

### 2.1. Catechin Labeling of Chitosan

Chitosan (Primex, Siglufjordur, Iceland; Mw: 130 kDa; DDA: 95%) was chemically labeled with catechin (Sigma-Aldrich, St. Louis, MO, USA; 98%) following the procedures described by Curcio et al. [28]. Accordingly, chitosan solution was prepared by dissolving 0.5 g in 50 mL of acetic acid solution (2%) under stirring for 2 h. Following this, 1 mL of 1 mM H_2_O_2_ with 0.054 g of ascorbic was included and the solution was stirred for another 30 min. Finally, 0.374 g of catechin was included with gentle stirring. The solution was stirred for 24 h at 22–23 °C. Subsequently, for purification, the solution was placed in dialysis tube (Millipore Corp., Burlington, MA, USA; MwCo 12,000–14,000 Da) and immersed in a 2 L flask filled with purified water. Dialysis was performed with gentle stirring of water at room temperature (22–23 °C) for 48 h, performing eight replacements of the water. The purified volume was then diluted to 1 mg/mL acetate buffer (pH 4.48 ± 0.01) used to dissolve the initial chitosan.

The amount of catechin, which was bound to chitosan, was determined by the amount of total phenolic equivalents using the Folin–Ciocalteu reagent procedure [30]. The modified chitosan was precipitated using 0.1 M NaOH solution and followed by filtration and washing with pure water and drying with a vacuum for at least 18 h. An amount of 20 mg was dissolved in 6 mL of water and 1 mL of Folin–Ciocalteu reagent (Sigma-Aldrich, St. Louis, MO, USA) was included under vortex mixing. Then, 3 mL of a 2% aqueous solution of Na_2_CO_3_ was included and the mixture was stirred for 2 h. Acquisition of absorbance was performed at 760 nm. A calibration curve was further constructed, reading the absorbance of solutions of pure catechin at 1 to 25 µg/mL. The total phenol content was obtained applying the equation of the standard curve of calibration of the solution, considering y as the absorbance at 760 nm with x as the total phenolic content. The calculated data unveiled that 23% of chitosan monomers were labeled with catechin. Analytical grade reagents were employed and pure water was obtained from MilliQ filter (Millipore Corp., Burlington, MA, USA) with organic carbon value of less than 15 ppb and 18 MΩ·cm resistivity.

### 2.2. Preparation of Composite Liposomes

Nano-scale vesicles, or liposomes, were prepared by the reverse-phase evaporation method [17,31]. This method is advantageous to allow for the coordination of chitosan to the lipid membrane. A total of 10 mg of 1,2-dioleoyl-*sn*-glycero-3-phosphocholine (DOPC), a phospholipid with two mono-unsaturated acyl chains between C9 and C10 (Avanti Polar Lipids, Alabaster, AL, USA; 99%), were used for each sample dissolved in chloroform and three different concentrations of chitosan–catechin dissolved in water slightly acidified with HCl solution (pH 5.3) were added with stirring, leading to samples with 0, 0.8, 1.6, 2.4 μM of polymer and all were prepared in duplicate. The mixtures were subjected to ultra-stirring (3 min) and then the solvent was evaporated with nitrogen gas stream. The samples were kept in a desiccator overnight under vacuum. Following this, 300 μL of aqueous solution of FeCl_2_ (0.5 mM) were added to each vial. The samples were submitted to ultrasonication (Benchmark Pulse 150 Ultrasonicator, Sayreville, NJ, USA; 10%, 2 min) to form nanovesicles. One series was challenged with oxidation with Fenton reaction by addition of 50 mM H_2_O_2_, immediate 1 min vortex mixing and maintaining the samples in the dark in a bath sonicator (Eco-Sonics, Sao Paulo, Brazil; 40 kHz, 110 W) at 37 °C, performing occasional 2 min sonication at every 4 h interval over a period of 24 h. The same procedure was performed for the equivalent series without hydroperoxide. Immediately after the procedure, all samples were analyzed for hydrodynamic size and zeta potential.

### 2.3. Preparation of Giant Vesicles

Giant vesicles (GUVs) without and with chitosan–catechin were obtained applying the electroformation method modified for inclusion of chitosan [18]. In brief, an amount of 10 μL of POPC, a phospholipid with two acyl chains, one being mono-unsaturated, between C9 and C10, in chloroform (2 mM) was placed over two conductive glass slides (with surface covered with indium tin oxide, ITO) for the preparation of lipid GUVs. For the preparation of GUVs with the lipid membrane covered with chitosan–catechin, a reverse phase emulsion was previously prepared by vortex mixing the lipid in chloroform including amounts of chitosan–catechin solution to prepare GUVs with three concentrations of polymer. The same amount of 10 μL of this emulsion was spread over ITO slides. All slides were dried with nitrogen gas stream and then kept in a desiccator under vacuum for 2 h. The slides were then placed with their conductive sides facing each other, separated by a spacer. This electro-swelling chamber was filled with 0.2 M sucrose solution and coupled to an alternating power generator (Gratten ATF20B 20 MHz, Jena, Germany) at a frequency of 1.5 V and 10 Hz for 2 h at room temperature (22–24 °C). Subsequently, the obtained GUV solution was placed in an Eppendorf vial. The procedure leaded to GUVs with three concentrations of chitosan–catechin, determined as previously described [18], of 0.9, 1.8 and 3.7 wt%, corresponding approximately to proportions of lipid to monomer of 100:8, 100:13 and 100:27. Microscopic observation was performed in an observation chamber built with two glass slides separated with a hollowed rubber spacer by mixing 50 μL of the GUV solution with 300 μL of a 0.2 M glucose solution. This created a sugar asymmetry between the inside and outside of the vesicles. Osmolarities of sucrose and glucose solutions were previously measured with an Osmomat 030 cryoosmometer (Gonotec, Berlin, Germany) and carefully matched to avoid osmotic pressure effects. The slight difference in density between the inner and outer solutions keeps the vesicles to the lower slide, where they can be easily observed on the inverted microscope, and in addition, the difference in refractive index between sucrose and glucose solutions provides a better contrast when observing the vesicles with phase contrast microscopy. For the experiments with oxidation of the lipid membrane, 40 μM of methylene blue (MB) photosensitizer was previously included in the glucose solution and the procedure described above was followed.

### 2.4. Preparation of Crystalline Cubic Phase

Bulk phase of crystalline cubic symmetry was produced with lipid monoolein (Sigma-Aldrich, St. Louis, MO, USA; 99%) and DOPC at the proportion 40:60 (*w*:*w*) containing different concentrations of chitosan–catechin. Lipids were dissolved in chloroform at a total concentration of 25 mg/mL; in 1 mL of this solution, chitosan–catechin solution (1 mg/mL) was added; different samples were prepared in duplicate with 0; 0.8; 1.6 and 2.4 μM of polymer. The mixtures were subjected to ultra-stirring and then the solvent was evaporated with nitrogen gas. All samples were kept overnight in a desiccator under vacuum. Following this, 300 μL of aqueous solution of FeCl_2_ (0.5 mM) was added to each vial. The samples were submitted to ultrasonication (Benchmark Pulse 150 Ultrasonicator, Sayreville, NJ, USA; 10%, 2 min: 3 s on, 3 s off) to form the bulk phase or hybrid hydrogel. One series was challenged with oxidation with Fenton reaction by addition of 50 mM H_2_O_2_, immediate 1 min vortex mixing and maintaining the samples in the dark in a bath sonicator (Eco-Sonics, Sao Paulo, Brazil; 40 kHz, 110 W) at 37 °C, performing occasional 2 min sonication at every 4 h interval over a period of 24 h. The same procedure was performed for the equivalent series without hydroperoxide. The samples were analyzed using SAXS after 18 h rest in the dark at ambient temperature (22–26 °C).

### 2.5. Dynamic Light Scattering and Zeta Potential

Quasi-elastic light scattering (QELS) and zeta potential acquisitions were performed using the Nano-ZS90 Malvern ZetaSizer (Malvern Instruments, Malvern, UK) operating with a 4 mW HeNe laser at 632.2 nm in a sample compartment at constant 25 °C. Amounts of 20 μL of liposomes dispersions were further diluted in purified water to 1 mL. The folded capillary zeta cuvettes were used and the scattered light was obtained at 173°. Exponential spacing of the correlation time was applied to obtain the autocorrelation function. The intensity-weighted size distribution was obtained by fitting data with a discrete Laplace inversion routine. Size determination was made using the Stokes–Einstein relation and the polydispersity was assessed by using cumulant analysis of the correlation functions measured by QELS applying the amplitude of the correlation function and the relaxation frequency. The second-order cumulant was used to determine the polydispersity of samples. Three consecutive measurements were performed for each sample.

Zeta potential was obtained for the same samples with 50 runs per sample. The electrophoretic mobility was converted to zeta potential using the Helmholtz–Smoluchowski relationship. The colloidal size was expressed as a hydrodynamic diameter in nm and zeta potential in mV.

### 2.6. Optical Microscopy

An inverted microscope Axiovert 135 (Carl Zeiss; Jena, Germany) with a Ph2 63× objective was used for the experiments with GUVs. The snapshots were acquired through a digital AxioCam HSm coupled camera (Carl Zeiss; Jena, Germany) in the transmission mode (bright field), using low illumination to observe the vesicles. With these parameters, any alteration of the vesicles in the presence of methylene blue occurred over 3 h under bright field. MB photoactivation was performed using a 103 W Hg lamp (HBO 50/AC) coupled to the microscope and the filter, λex = 665 nm; λem = 725 nm.

### 2.7. Small-Angle X-ray Scattering

SAXS experiments were performed on SAXS1 beamline of the Brazilian Synchrotron Light Laboratory (LNLS, Campinas, Brazil). The liquid crystalline samples were individually placed, with the use of a spatula, in-between two mica slides separated by a gap of 1 mm. Temperature during acquisitions was constant at 25 °C and 60 s exposure per sample was performed. Scattering data were obtained with a 2D Pilatus 300 K detector, with pixel sizes 172 μm × 172 μm, located at 1 m from the sample holder. According to the standard procedures, the obtained data were normalized to beam intensity and time, and corrected for transmission, sample thickness, parasitic and background scattering. The results were converted into absolute units (cm^−1^) by using the absolute scattering cross-section of water as an internal standard. The procedure provided SAXS profiles as a function of scattering vectors in the *q* range 0.13–4.90 nm^−1^, where *q* = 4π/λ × (sin θ), λ is the wavelength of the incident X-ray photons (λ = 1.55 Å) and θ is the scattering angle. The *q*-range corresponds to length scales between ~2 and ~50 nm. Therefore, SAXS data provide information of the internal structure of the liquid crystalline samples.

The lattice parameters (*a*) of the liquid crystalline phases were obtained from the Bragg peaks acquired in the X-ray diffraction patterns. The respective reflections were fitted through the Miller indexes applying the following equation:

*q* = (2π/*a*) (*h*^2^ + *k*^2^ + *l*^2^)^1/2^ (1)

from linear fits plotting *q* versus (*h*^2^ + *k*^2^ + *l*^2^)^1/2^, where *q* is the peak position along the scattering vector axis and *h*, *k* and *l* are the Miller indices of the corresponding cubic lattice. The slope of the linear fit to the data equals the inverse of the cubic lattice parameter.

### 2.8. Cell Culture and Cytotoxicity Assay

To evaluate the potential cell toxicity of chitosan labeled with catechin (CHCa), a cytotoxicity experiment was conducted on HeLa cells using the MTT assay. Initially, the cells were seeded at a density of 10,000 cells per well in a 96-well plate, each well containing 100 µL of Dulbecco’s Modified Eagle’s Medium (DMEM High Glucose; Thermo Fisher Scientific, Waltham, MA, USA) supplemented with 10% fetal bovine serum (FBS, Thermo Fisher), 1% GlutaMAX™ (Thermo Fisher), and 1% penicillin/streptomycin (Thermo Fisher). After incubation for 24 h at 37 °C in a humidified incubator with 5% CO_2_, CHCa was added to the cells. The CHCa samples, initially prepared at a concentration of 146.70 µM in acetic acid (HAc: 175 mM, Synth, São Paulo, Brazil), were serially diluted, and 10 μL of these solutions was added to each well (concentrations are detailed in Table S1). As a control group, HAc was used in an equivalent serial dilution without CHCa (refer to Table S2). At a timepoint 24 h post-treatment, the medium was replaced and 10 μL of MTT solution (3-(4,5-dimethyl-2-thiazolyl)-2,5-diphenyl-2H-tetrazolium bromide, 5 mg/mL in phosphate-buffered saline solution; Sigma-Aldrich, St. Louis, MO, USA) was added to each well, and the plate was incubated at 37 °C for 4 h in a light-protected environment. Subsequently, the MTT-containing medium was aspirated, and the resultant formazan crystals were solubilized with 100 μL of Dimethyl sulfoxide (DMSO: Sigma-Aldrich, St. Louis, MO, USA), with constant stirring for 10 min under light-protective conditions. The absorbance of the dissolved formazan was measured at 570 nm using a SpectraMax M2 microplate spectrophotometer (Molecular Devices, San Jose, CA, USA). Cell viability was determined by comparing the results with a calibration curve constructed with unexposed cells, considering the viability of control cells as 100%. Two independent experiments were conducted, each in triplicate.

### 2.9. Statistical Analysis

Statistical analysis was performed using GraphPad Prism 8.0.2 (Boston, MA, USA). The values are expressed as the mean ± standard deviation (SD). Mean values were compared using one-way ANOVA, unless indicated otherwise. Differences were considered statistically significant at *p* < 0.05.

## 3. Results and Discussion

### 3.1. Cytotoxicity of Chitosan–Catechin

Before evaluating the antioxidant protection capacity of the flavonoid-labeled biopolymer on the lipid membranes, the cytotoxicity of the same was evaluated, aiming to disclose the applicability potential of the material in terms of the cell safety. To this aim, we used the MTT assay, which is a widely used method for assessing cell viability and proliferation based on the metabolic activity of cells. The MTT assay measures the reduction of a yellow tetrazolium salt, MTT, to a purple formazan product by the mitochondrial dehydrogenases present in living cells. The produced formazan crystals can be dissolved in DMSO, allowing for the absorbance of the resulting solution to be quantified using a spectrophotometer. Consequently, this assay is used to assess the metabolic rate, which is indicative of the cell viability. To perform this experiment, HeLa cells were chosen because they have been widely employed in biomedical research for decades on a variety of topics such as cancer, cell biology and genetics, as well as general models for in vitro drug delivery and toxicity studies [32,33].

**Figure 1:**
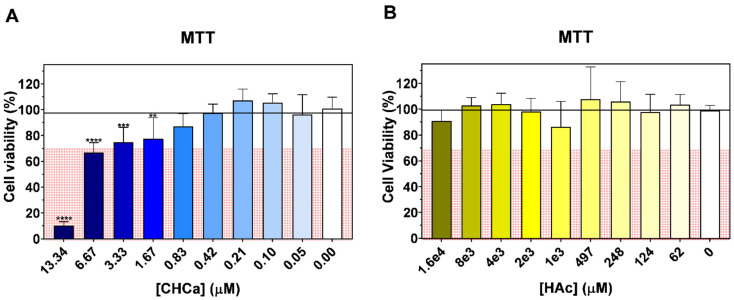
Assessment of HeLa cell viability via MTT assay following CHCa (**A**) and HAc (**B**) treatment. Two independent experiments were carried out, each performed in triplicate. One-way ANOVA was used to compare different concentrations of CHCa and HAc with control group (0 mg/mL or mM, respectively). ** *p* < 0.005, *** *p* < 0.0005 and **** *p* < 0.0001.

The cytotoxicity assessment of the CHCa using the MTT assay revealed dose-dependent effects. According to the standards set by ISO 10993-5:2009 [34], the CHCa exhibited significant toxicity in the HeLa cells at concentrations above 6.67 µM, resulting in a cell viability lower than 70%. However, at concentrations below 3.33 µM, the CHCa falls into the non-toxic category, considering it safe for use (Figure 1A).

In parallel, the HAc concentrations used in the preparation of the CHCa were assessed for cell viability. The results indicate that none of the tested HAc concentrations adversely affected the cell viability over 24 h, (Figure 1B), indicating that the solvent used in the CHCa preparations is non-toxic to HeLa cells. Consequently, all the concentrations of CHCa and HAc utilized in this study are considered non-toxic.

### 3.2. Photo-Oxidation of Giant Vesicles

**Figure 2:**
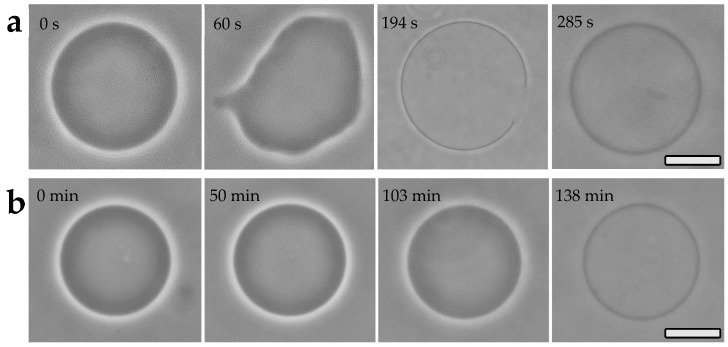
Snapshots of microscopy images in the phase contrast mode of characteristic time sequences of (**a**) the damage on POPC giant vesicles membrane by photoactivation of MB (665 nm; 40 μM) from left to right: stable vesicle right before MB irradiation; vesicle flickers, increase in membrane surface and bud release; phase contrast decay and large transient pore take place; complete loss of phase contrast; (**b**) GUV covered with chitosan–catechin (1.8 wt%) showing higher membrane stability and prolonged phase contrast decay with MB irradiation. Start time: start of Hg lamp on the microscope. Bars span 20 μm for all.

The irradiation of methylene blue (MB) dispersed in an external solution of GUVs has been shown to promote substantial changes in lipid membranes [29,35]. As shown as an example in Figure 2a, the initial spherical shape of the GUVs is affected by large fluctuations associated with surface area increase, which leads to membrane reshaping. In the sequence, the GUV spherical shape is recovered, and the excess of area usually is released by filaments and/or bud emission [36], followed by the formation of relatively large transient micropores on the membrane and then the loss of the initial phase contrast. Such an effect is due to the exchange of sucrose/glucose solutions from internal and external media, respectively, due to the formation of pores in the membrane (Figure 2a). Of note, fading contrast can also be observed with no micropore formation. This means that the presence of nanopores and/or membrane defects caused by photo-oxidation, and not seen by optical microscopy, can also allow for sugar exchange [29,37].

It has been previously described that the formation of POPC hydroperoxides (POPC-OOH), as a result of singlet oxygen action (produced by MB photo-irradiation) on the double bond of the lipid acyl chain, is responsible for the increase in membrane area, as seen in Figure 2a. This results in membrane reshaping with the subsequent formation of buds and/or membrane protrusion to release the area excess [29,36]. It is worthy of note that when singlet oxygen is the only oxidizing agent, hydroperoxides are the sole product of lipid oxidation [37]. However, hydroperoxides may also act as a substrate for additional reactions by excited photosensitizers, allowing the lipid peroxidation propagation that ends up in truncated oxidized chain lipids [37]. This indeed requires close contact between the excited photosensitizer and the substrate. Interestingly, previous experimental liposomes photo-damaged by MB irradiation has revealed the formation of truncated lipid aldehydes in amounts as low as 1 mol%, which correlates with membrane permeability increase [38]. Therefore, the phase contrast fading here observed after a period of time (Figure 2a) may be associated with the formation of oxidized truncated chain lipids in the continuity of the lipid photo-oxidation by a direct reaction between the excited MB triplet state and the hydroperoxides generated by singlet oxygen action on the lipid unsaturations. It should be remarked that, in a previous study, we reported that unmodified chitosan over the membrane of POPC GUVs was not able to protect the lipids, hence the vesicles quickly collapsed following the irradiation of MB dispersed in the outer GUV solution [29].

On the other hand, in the presence of chitosan–catechin, the GUVs did not show significant fluctuation or modification during at least 30 min of continuous photo-irradiation of the GUV solutions containing MB (Figure 2b). An effective interaction of chitosan with the membrane of the GUVs was previously observed using a fluorescent probe on the polymer, and the fluorescence microscopy images have confirmed the location of chitosan over the lipidic membrane [29,39]. In the present study, the protected GUVs remained microscopically stable under in situ photo-oxidation, since the increase in the membrane surface area, as well as the formation of large micropores, were avoided.

**Figure 3:**
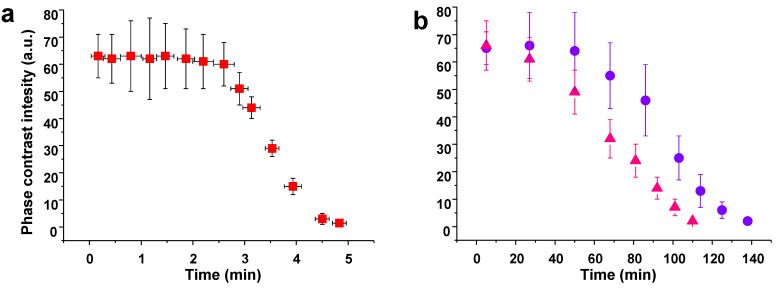
Profiles of phase contrast fading over time for POPC giant vesicles (**a**) and the same containing chitosan–catechin (**b**) at 0.9 (▲) and 1.8 wt% (●) submitted to photoactivation of MB (665 nm; 40 μM). Each point was obtained from the average of at least 10 vesicles and the uncertainty is shown by the error bar.

The fading of phase contrast, reported as the solution traffic through the pores on the vesicle membrane, was largely delayed for the GUVs containing chitosan–catechin over the membrane. Therefore, we evaluated the loss of contrast over time for individual GUVs by the integration of the gray level profiles over the vesicle membranes in the phase contrast microscopy images, as previously described [29]. The results are shown in Figure 3. 

The first curve (Figure 3a) corresponds to the sample with GUVs of pure lipid, revealing that contrast decay starts a little after 2.5 min of continuous MB photo-irradiation and the complete loss of phase contrast occurs within, approximately, a further 2 min, showing a relatively fast optical contrast decay. This result confirms that the oxidative process initiated by singlet oxygen and mediated by a contact-dependent mechanism between the excited MB triplet state and the hydroperoxidized lipid [37] effectively leads to the permeability increase in the vesicles’ membranes. Now, comparing this to the results for the GUVs containing chitosan–catechin over the membrane (Figure 2b and Figure 3b), it is evidenced that phase contrast decay started only after 27 min for the lower polymer concentration reaching completeness around 110 min. For the intermediate polymer concentration, the processes started after 50 min and extended to about 140 min. Furthermore, considering the higher polymer concentration, no significant phase contrast change was found during 3 h of the continuous photo-irradiation of MB indicating a remarkable membrane photo-protection. 

**Scheme 1:**
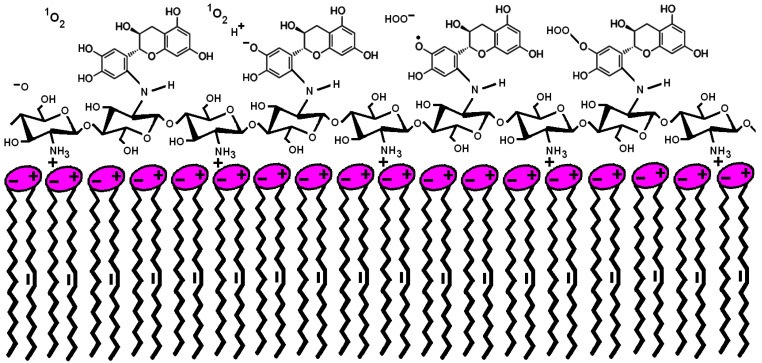
Coordination of chitosan–catechin segment over a layer of phospholipids by means of known electrostatic interaction [40] and mechanism of singlet oxygen scavenging by O-H dissociation at phenolic ring of chemically bonded catechin depicting the antioxidant activity of the flavonoid. The biopolymer provides a protecting shield on lipids membranes.

Therefore, the results evidence that the inclusion of chitosan–catechin over the membrane of the GUVs, the coordination of which has been described as mainly governed by electrostatic interactions [40], provides a kind of protecting shield on the POPC lipids (Scheme 1), which indeed delayed or avoided the formation of pores/defects that allow for solution traffic through the membrane, which occurs in absence of the antioxidant biopolymer (Figure 2a). We suggest that the chitosan–catechin layer over the membrane works as a physical barrier that hinders the diffusion of MB from the solution to the membrane surface, thus avoiding the contact between the excited photosensitizer and the lipids. Additionally, as proposed in Scheme 1, the singlet oxygen generated by MB photoactivation is also scavenged by the catechin phenolic O-H dissociation, being an electron transfer process, by which the phenol is converted into a phenoxyl radical, responsible for the scavenging of singlet oxygen [41]. Moreover, positioning the flavonoid catechin as the singlet oxygen scavenger right over the membrane surface strikingly lessens the increase in the membrane area, owing to the alleviation of photo-generated lipid hydroperoxides. Altogether, the generation of oxidation byproducts which could follow the hydroperoxidation of lipid and promote the permeability and emergence of large pores is actually avoided by this strategy of membrane protection.

### 3.3. Liposome Structural Stability

Table 1 shows the results of the structural parameters of hydrodynamic size and zeta potential for the different samples of liposomes with varying concentrations of antioxidant polymer without and with oxidative solution. The known Fenton reaction produces a hydroxyl radical in the solution, which can react with the unsaturated acyl chains of the lipid DOPC on liposomes membrane:

Fe^2+^ + H_2_O_2_ → Fe^3+^ + OH^−^ + OH·

The results show that the samples without polymer and with the lower concentration of polymer have endured significant changes in the oxidative solution, with the decomposition of the initial nanovesicle, as shown by the formation of two distinct populations of hydrodynamic sizes, as well as the two results for the zeta potential. These results suggest that DOPC has undergone at least partial oxidation which has possibly led to the production of hydroperoxide as well as new chemical structures as a result of the lipid acyl chain cleavage. In this sense, Kunimoto et al. [42] have described for phosphatidylcholine liposomes in iron solution the formation of oxidation byproducts due to lipid breakdown. The formation of water-soluble byproducts was reported along phosphorous-containing products, one of which was characterized as lysophosphatidylcholine. The permeability increase in these liposomes was associated with the accumulation of peroxidized products in the membrane. In our study, the formation of oxidation products must have resulted in the rupture of some of the liposomes membrane. Hence, an aggregation process may have resulted from partial liposomes collapse connecting remaining non collapsed liposomes, thus providing an increase in the size. On the other hand, the reduced size of the population must be associated with the smaller vesicles produced with the oxidized lipids of conical shape, such as lysophosphatidylcholine, which provide a higher membrane curvature, allowing the formation of small vesicles [43].

In the case of the samples with polymer, the lowest concentration also showed destabilization, suggesting that 0.8 μM of biopolymers was not sufficient to effectively protect the vesicles’ structures. However, considering the intermediate and higher chitosan–catechin concentrations, the results evidence that no significant changes occurred in the size or even in the zeta potential. Therefore, the size and zeta alterations of the effectively oxidized liposomes must actually be reported by the oxidation process of the DOPC, as previously described for GUVs [29], leading to the disruption of the vesicles and formation of new structures and aggregates, as discussed above.

Of note, the high values of the zeta potential of all the samples must be related to the Fe^2+^ coordination to the vesicles’ surfaces. Considering the data in Table 1a, the significant increase in the zeta potential of the liposomes containing the biopolymer, compared to the pure lipid liposome, evidences that the iron ions’ coordination to the surface of the nanovesicles is highly increased in the presence of chitosan–catechin over the membrane. Additionally, a slight decrease in the average values of the zeta potential in the oxidative solution is noticeable for the liposomes with 1.6 and 2.4 μM chitosan–catechin. This may be related to the oxidation of Fe^2+^ to Fe^3+^ [44], suggesting that the coordination of Fe^3+^ on the composite liposomes may be reduced in comparison to Fe^2+^.

Overall, the results show the effective structural protection of the liposomes provided by the chitosan–catechin over the membrane at 1.6 and 2.4 μM, that may be related to the oxygen radical inactivation power of the flavonoid chemically linked to the polysaccharide. From a molecular perspective, it is known that the antioxidant properties of phenolic structures may be reported as the phenoxyl resonance, the steric hindrance of bulky groups that replace hydrogen in the aromatic ring and the dissociation of the OH bond [41]. For chitosan–catechin, it can be considered that a hydrogen in the aromatic ring of catechin is substituted in the binding with the chitosan backbone, also providing a secondary amino bound with it. Thus, in this new molecular arrangement, the steric hindrance to the approach of an oxidizing agent is increased. Furthermore, the molecular structure of chitosan, which contains several oxygen groups on its monomers, is also an effective electroactive complex for the phenoxyl radical. These contributions enhance the delocalization of the phenoxyl radical resonance in the catechin and the dissociation of OH bonds, thus increasing the electron density and the power of neutralizing oxygen radicals, thereby providing efficient antioxidant protection to the lipids of the liposome membrane.

### 3.4. Liquid Crystalline Topology

Monoolein is a lipid with a single alkyl chain well known for its property of self-assembling crystalline topologies in varying conditions of temperature and solvent composition [45]. Hence, monoolein bulk phases comprise long-range water labyrinths cloistered between bicontinuous lipid bilayers displaying specific curvatures. In this way, cubic phases may be produced in lipid/water systems in the regions lying between the transition of the liquid crystalline lamellar phase and the inverse hexagonal H_II_ phase, and they usually take place in narrow ranges of temperature and/or a strict proportion of components [46,47,48]. In excess water and room-temperature monoolein, with its cone-like molecular shape, assembles as a bulk phase of Pn3m cubic symmetry, which by turn, can transition to new symmetries in the presence of additives, such as other lipids of specific molecular shapes and different critical packing parameters [45,48].

**Figure 4:**
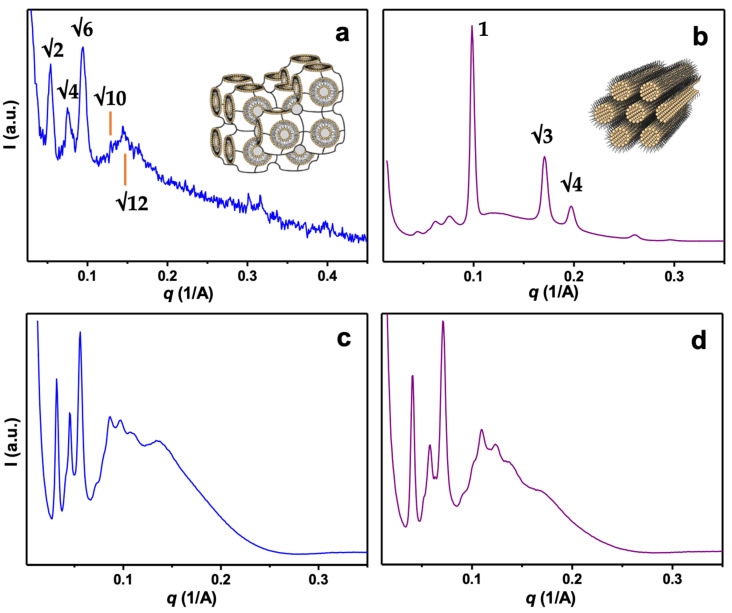
SAXS patterns of monoolein/DOPC (40/60 *w*/*w*) liquid crystalline phase (**a**), the same in oxidative solution (**b**), hybrid lipid–biopolymer phase (0.8 μM chitosan–catechin) (**c**) and the same in oxidative solution (**d**). The indexing of the Bragg peaks is shown in upper panels along the respective models for cubic phase of Im3m space group and inverse hexagonal H_II_.

In this study, monoolein was mixed with DOPC, which is a phospholipid of two alkyl chains and is cylindrically shaped, thus providing means to change the curvature of the lipid bilayer in the bulk phase [48]. The SAXS results shown in Figure 4a evidenced a prominent Im3m cubic lattice symmetry with the Bragg peaks spaced in the ratios √2:√4:√6:√10:√12, unveiling that the inclusion of DOPC provided an effective phase transition of the characteristic original Pn3m cubic phase of bulk monoolein. However, by submitting this Im3m phase to the oxidative media of a Fenton reaction, a remarkable liquid crystalline phase change was unveiled. As shown in Figure 4b, new peaks of significant higher intensity appeared in the SAXS profiles. These new peaks index in the ratios 1:√3:√4, denoting the formation of an inverse hexagonal H_II_ phase, for which the bilayer displays a negative mean curvature towards the aqueous interior, thus providing cylindrical water channels. Thereby, the oxidation process of the lipids effectively leads to a change in the bilayer curvature [49,50], resulting in the transition to a new liquid crystalline topology. Despite this, the original Im3m cubic phase also has relatively lower Bragg peaks before and between the ones of high intensity corresponding to the H_II_ phase. This result further proves that the bulk material presents as a partial cubic and partial inverse hexagonal structure, suggesting that not all the lipids in the bulk were oxidized. Nevertheless, considering the significant higher intensity of the H_II_ reflections, one may speculate that the majority of the oxidized monoolein/DOPC bulk phase comprises a H_II_ structure. Hence, the oxidation process provided a transition from the 3D to the 2D crystalline order.

Nevertheless, the results for the hybrid lipids and chitosan–catechin “hydrogels” have shown important peculiarities. As shown in Figure 4c, well-defined Bragg peaks, which also index in the ratios √2:√4:√6:√10:√12, evidence a defined Im3m cubic lattice. The same result was found for all three of the concentrations of the chitosan–catechin. Hence, the introduction of biopolymer did not alter the crystalline symmetry, although the internal distances of the crystalline structure have been slightly increased (Table 2).

Through subjecting the composite lipid–biopolymer phase to the oxidative media, the SAXS results show that the Bragg reflections have kept the same indexing; thus, the Im3m cubic lattice was indeed preserved (Figure 4d). In contrast to the lipid phase, no signs of the H_II_ phase, nor other cubic or lamellar phase, were identified for all the samples containing chitosan–catechin. This result evidences once more that the antioxidant biopolymer was effective in preventing structural changes in the lipid bilayer that could result from oxidation provided by the Fenton reaction. 

Notwithstanding, some alterations in the lattice parameter (*a*) of the crystalline structure were identified as shown in Table 2. Firstly, *a* increased systematically with the inclusion and concentration of the chitosan–catechin. This result is expected, considering that the assembly of the cubic network incorporates a relatively large macromolecule. A similar result has been previously described for multilamellar liposomes modified with chitosan [16], lipid–chitosan hydrogel [19] and for cubosomes tailored with biopolymers [20]. Secondly, for the hybrid lipid–biopolymer phase, *a* decreased for the oxidation-challenged samples. Hence, despite the absence of a phase transition, the reduction in *a* for the samples with chitosan–catechin denotes an additional structural effect of the oxidative media, which may be related to the lower extent of lipid oxidation and/or the dehydration of the crystalline network, i.e., a reduction in the water labyrinths [20,51]. Although, this structural change is notably to a lesser extent when compared to the phase transition to the H_II_ phase that occurred in absence of the biopolymer. Therefore, it is evidenced that chitosan–catechin provided noteworthy structural stability to the cubic network in the oxidative media.

## 4. Conclusions

Oxygen radicals react with lipids leading to severe structural alterations in vesicles produced with lipid bilayers. Chitosan labeled with catechin flavonoid was produced to be applied as an antioxidant component to prevent lipid oxidation. A cell toxicity assay unveiled the safety of chitosan–catechin for the applied range of concentrations and evidenced the modified biopolymer as a non-toxic material. The antioxidant protection was effective in studying the physical stability of the three vesicular systems, i.e., liposomes, giant vesicles and liquid crystalline cubic phase, where the biopolymer was incorporated into the structure. In the giant vesicles, our studies with the chitosan–catechin antioxidant macromolecule showed effective membrane protection against the oxidation effects generated by methylene blue photo-irradiation. For the nanovesicles, the size and zeta potential of the liposome evidenced degradation of the structure by hydrogen peroxide and hydroxyl radicals generated by the Fenton reaction, which was minimized in the lipid/chitosan–catechin composite liposome. Finally, the production of a lipid/chitosan–catechin composite crystalline phase revealed a specific cubic topology, which was preserved in the strong oxidative media. Moreover, when applying the nanovesicles made with lipids in the biological media, the lipidic vesicle membrane will face harsh physical and biochemical conditions, e.g., in the gastrointestinal tract, thus altering the membrane curvature and promoting lipid oxidation. In the presence of chitosan–catechin on the membrane, the study showed antioxidant protection for the lipidic membranes of different curvatures, i.e., the giant vesicles of very low curvature, liposomes of medium membrane curvature, and cubic phase of high membrane curvature. Hence, the antioxidant effect was also supplied for the different membrane curvatures. Therefore, an efficient antioxidant strategy was afforded to the different vesicular systems, hence protecting the lipid membranes and providing structural stability to the micro- and nanovesicles, that can be potentially applied for the protection and delivery of diverse bioactive and nutraceutical compounds.

## Figures and Tables

**Table 1:** Hydrodynamic diameters (Dh) and zeta potential of liposomes with different concentrations of chitosan–catechin (CHCa) before (a) and after (b) challenging with Fenton reaction.

	(a)		(b)	
CHCa (μM)	Dh (nm)	Zeta (mV)	Dh (nm)	Zeta (mV)
**0**	84 ± 8	+42.9 ± 3.4	49 ± 12	+73.7 ± 6.5
			(20%)	(17%)
			252 ± 24	+48.1 ± 7.3
			(80%)	(83%)
**0.8**	96 ± 9	+65.4 ± 5.6	34 ± 10	+75.5 ± 7.6
			(27%)	(21%)
			268 ± 18	+44.8 ± 8.1
			(73%)	(79%)
**1.6**	103 ± 11	+66.7 ± 4.9	110 ± 11	+52.4 ± 5.1
**2.4**	105 ± 9	+63.5 ± 5.3	117 ± 9	+55.7 ± 5.9

All variations (±) are standard deviations obtained from the respective experiment.

**Table 2:** Liquid crystalline phase symmetry and lattice parameter (*a*, nm) obtained with SAXS for lipid–biopolymer phase with increasing concentration of chitosan–catechin (CHCa, μM) before (a) and after (b) Fenton oxidation.

	(a)		(b)	
CHCa	Symmetry	*a*	Symmetry	*a*
**0**	Im3m	14.7	H_II_/Im3m	8.5/17.7
**0.8**	Im3m	26.7	Im3m	21.8
**1.6**	Im3m	27.4	Im3m	23.6
**2.4**	Im3m	27.9	Im3m	25.3

## Data Availability

We declare that our research data are available on reasonable request.

## Supplementary Materials

The following supporting information can be downloaded at https://www.mdpi.com/article/10.3390/pharmaceutics16010141/s1, Table S1 and Table S2, concentrations of chitosan–catechin and acetic acid used in the cell culture and cytotoxicity assay.
