# Supplementary material for: Flavonoid-Labeled Biopolymer in the Structure of Lipid Membranes to Improve the Applicability of Antioxidant Nanovesicles
Source: Pharmaceutics. 2024 Jan 20;16(1):141. doi: 10.3390/pharmaceutics16010141 (PMC10819309; doi:10.3390/pharmaceutics16010141)
Supplement: Supplementary file 1 [file pharmaceutics-16-00141-s001.zip › pharmaceutics-2800434-supplementary.pdf]

Supporting Information for:

Flavonoid-Labeled Biopolymer in the Structure of Lipid Membranes to Improve the Applicability of Antioxidant Nanovesicles

Patrick D. Mathews [1,2], Gabriella S. Gama [1], Hector M. Megiati [1], Rafael R. M. Madrid [1], Bianca B. M. Garcia [3], Sang W. Han [3], Rosangela Itri [4] and Omar Mertins [1,*]

[1] Laboratory of Nano Bio Materials (LNBM), Department of Biophysics, Paulista Medical School, Federal University of Sao Paulo, Sao Paulo 04023-062, Brazil; patrick.mathews@unesp.br (P.D.M.); gabgamasantos@gmail.com (G.S.G.); hectormegiati@gmail.com (H.M.M.); rafael.madrid@unifesp.br (R.R.M.M.)
[2] Institute of Biosciences, Sao Paulo State University, Botucatu 18618-689, Brazil
[3] Interdisciplinary Center for Gene Therapy, Paulista Medical School, Federal University of Sao Paulo, Sao Paulo 04023-062, Brazil; bianca.bonetto@unifesp.br (B.B.M.G.); sang.han@unifesp.br (S.W.H.)
[4] Applied Physics Department, Institute of Physics, University of Sao Paulo, Sao Paulo 05508-900, Brazil; itri@if.usp.br
* Correspondence: mertins@unifesp.br

Table S1: CHCa concentrations. Sample C1 was initially prepared in HAc 175 mM, and subsequent dilutions were made in OPTI-MEM.

	C1	C2	C3	C4	C5	C6	C7	C8	C9
[Inicial] (µM)	146.70	73.35	36.68	18.34	9.17	4.58	2.29	1.15	0.57
V (µL per well)	10.00	10.00	10.00	10.00	10.00	10.00	10.00	10.00	10.00
Vf well (µL)	110	110	110	110	110	110	110	110	110
[Final] (µM)	**13.34**	**6.67**	**3.33**	**1.67**	**0.83**	**0.42**	**0.21**	**0.10**	**0.05**

Table S2: HAc concentrations. Sample HAc1 was initially prepared in water, and subsequent dilutions made in OPTI-MEM.

	HAc1	HAc2	HAc3	HAc4	HAc5	HAc6	HAc7	HAc8	HAc9
[Inicial] (mM)	175.00	87.50	43.75	21.88	10.94	5.47	2.73	1.37	0.68
V (µL per well)	10.00	10.00	10.00	10.00	10.00	10.00	10.00	10.00	10.00
Vf well (µL)	110	110	110	110	110	110	110	110	110
[Final] (µM)	**1.6E+04**	**8.0E+03**	**4.0E+03**	**2.0E+03**	**1.E+03**	**497.16**	**248.58**	**124.29**	**62.14**
